# Hybrid composite of Nafion with surface-modified electrospun polybenzoxazine (PBz) fibers *via* ozonation as fillers for proton conducting membranes of fuel cells

**DOI:** 10.1039/d2ra00830k

**Published:** 2022-03-25

**Authors:** Ronaldo P. Parreño, Arnel B. Beltran

**Affiliations:** Chemicals and Energy Division, Industrial Technology Development Institute (ITDI), Department of Science and Technology (DOST) Taguig 1631 Philippines rpparreno@itdi.dost.gov.ph rpparrenojr@yahoo.com; Department of Chemical Engineering, De La Salle University 2401 Taft Avenue Manila 1004 Philippines arnel.beltran@dlsu.edu.ph; Center for Engineering and Sustainable Development Research, De La Salle University 2401 Taft Avenue Manila 1004 Philippines

## Abstract

Nafion was investigated for its compatibility in the preparation of hybrid composites with electrospun Polybenzoxazine (PBz) surface-modified fibers by evaluating the effects on the surface and structure of the composite. A PBz fiber mat was first crosslinked by thermal treatment after electrospinning to enhance the mechanical integrity of the fibers prior to modification. Further surface modification *via* free radical ozonation was carried out by potentiating oxygen-based functional groups of hydroxyl radicals (–OH) onto fibers' exposed surfaces. The sequential modifications by crosslinking and ozone treatment were evaluated by analyzing surface properties using XPS, ATR-FTIR and water contact angle which determined the enhanced properties of the fibers that were beneficial to the target functionality. Electron spectroscopy confirmed that fibers' surfaces were changed with the new surface chemistry without altering the chemical structure of PBz. The presence of higher oxygen-based functional groups on fibers' surfaces based on the resulting atomic compositions was correlated with the change in surface wettability by becoming hydrophilic with contact angle ranging from 21.27° to 59.83° compared to hydrophobic pristine PBz fibers. This is due to electrophilic aromatic substitution with hydroxyl groups present on the surfaces of the fibers endowed by ozonation. The resulting surface-modified fiber mat was used for the preparation of composites by varying two process parameters, the amount of Nafion dispersion and its homogenization and curing time, which was evaluated for compatibility and interaction as fillers to form hybrid composites. The analyses of SEM images revealed the effects of shorter homogenization and curing time on composites with rougher and wrinkled surfaces shown on the final hybrid composite's structure while decreasing the amount of Nafion at the same homogenization time but longer curing time showed its influence on improvement of compatibility and surface morphology.

## Abstract 2

Nafion compatibility in the preparation of hybrid composites with electrospun Polybenzoxazine (PBz) surface-modified fibers *via* ozonation by evaluating the effects on the surface and structure of the composite.

## Graphical abstract

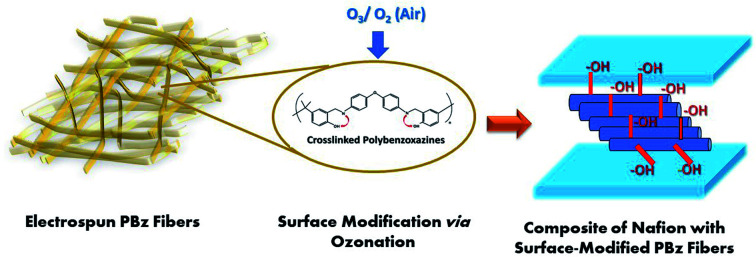

## 1. Introduction

Polymer electrolyte membrane fuel cell (PEMFC) is one of the most promising type of fuel cells, but currently still need further enhancement and innovation to reduce cost, improve durability and optimize performance.^1^ PEMFCs are currently operated at relatively low temperature (50–80 °C) which is causing limitation in terms of commercial viability.^2^ With these technical challenges, it has been proven that low temperature operating conditions for PEMFCs have several disadvantages which are the main reasons for not commercializing it yet on a larger scale. All of these problems can be overcome by operating it at relatively high temperature (>80 °C).^3^ This entails further innovative approaches on a unit cell-level to overcome the technological issues on cost and performance of the membrane component.^4,5^ Material selection, development and improvement are the important aspects of technological advancement for improvement of the fuel cell membrane's performance and reduction in cost.^6^

Currently, the proton conducting membrane used in PEMFC is the perfluorosulfonic acid (PFSA) membrane or commercially known as Nafion, which is the current standard for electrolyte of PEMFC up to the present time.^2,5,7^ It is considered as the standard polymer membrane for fuel cell due to its thermal and chemical stability as well as high ionic conductivity.^7–9^ These notable features of Nafion were the results of its structure composed of tetrafluoroethylene (TFE) backbone and perfluorovinly ether terminated with sulfonate groups.^5,8^ The structure produced a hydrophobic phase from the TFE and a hydrophilic phase of sulfonic acid group which acts as a reservoir for water during transport of protons.^3^ The water promotes the dissociation of protons from the sulfonic acid groups and provides highly mobile hydrated protons.^3^ Thus, it is essential for Nafion to be fully hydrated for good proton conductivity.^7,8^ Although Nafion is still the commercially used membrane, the problems remain unresolved to make it appropriate for long term operation under more severe conditions without losing its conductivity and stability that will reduce system costs.^10^ Increasing the temperature (>80 °C) at low relative humidity will affects its performance due to anisotropic membrane swelling which results to irreversible conductivity decay.^3,11^ At this condition, Nafion has weaker proton conductivity performance due to dehydration of the initial ionic domains.^8^ This results to lower power output as well as reduced mechanical strength.^12^

There were several investigations conducted on improving Nafion by adding functional additives, but in most cases the proton conductivity is lower than pristine Nafion membrane.^13^ In addition, research efforts in the past were focused on the modification of Nafion to address its weaknesses at high temperature and low humidity. These studies were conducted to improve its water management by incorporating hygroscopic components and high conductivity fillers such as silica and inorganic materials.^14^

Recent development in composite membrane involves incorporating nanofibers produced from electrospinning method.^9,13^ The concept of fiber-reinforced polymeric composite is very simple which combines the fiber and the polymer material with properties limited to those of the two components but gained a wide range of applications in aerospace, marine, civil engineering, energy, medicine, electronics and other emerging scientific domains.^15^ Incorporation of fibrous structures of nanostructured materials into a polymer matrix is now widely investigated as potential strategy for the development of proton-conducting membrane. Electrospun nanofibers of a proton-conducting polymer embedded in an inert polymer can provide pathways for proton conduction while the inert polymer will serve as the mechanical support.^9^ This made composting a viable approach for developing membrane to possibly overcome the limitations of perfluorinated and non-fluorinated hydrocarbon polymers. Composite has the main advantage of availability of diverse materials that can be used in various forms through different fabrication methods.^7^ It has been established that combining dissimilar organic and inorganic properties within a single material to form a composite produces superior properties compared to their pure components.^7,16^

In this study, electrospun fibers of polybenzoxazines (PBz), a high-performance thermoset polymer,^17^ were modified for its surface properties *via* free radical ozonation treatment and were utilized as fibrous fillers for the preparation of hybrid composite with Nafion. Electrospun fibers used in the preparation of composite as reinforcing materials have proven their benefits from involvement of more than one component due its various physical, structural, and chemical properties resulting to new materials.^18^ The fiber mat used was fabricated by electrospinning process which is known to have beneficial effects on morphology and surface properties.^18^ Nafion composite with polymer fibers could possibly reduce the humidity-induced stress but also improve the water transport under low or dry condition.^19^ Through this theoretical assumption, the modification *via* ozone treatment was explored in this work to investigate the effect of oxygen-based functional groups of hydroxyl radical (–OH) potentiated onto fibers' exposed surfaces. This composite preparation with surface-modified non-woven fibers is a potential innovation in finding solution to water transport and hydration of Nafion. But the compatibility of PBz surface-modified fibers in the preparation of composite with Nafion was the preliminary consideration for hybridization, which to the best of our knowledge, was the first time such an approach has been used for crosslinked and ozonated PBz fibers. Thus, this study looked into the possible benefits of employing simple preparation method for hybrid composite by only varying significant process parameters. The relationships between the amount, homogeneity and curing time of Nafion prior to combining with fibers in hybrid composite preparation were examined as potential strategy for the development of proton-conducting membrane. Through this study, the development of hybrid composite with Nafion involving two important aspects, surface modification of fibers as fillers and blending the polymer-fibers, was evaluated for compatibility by analyzing the surface structure and interaction in forming the composite.

## 2. Experimental section

### 2.1 Materials

Polybenzoxazines (PBz) was prepared in the lab as reported in the work of Lin *et al.*^20^ Dimethylsulfoxide (DMSO) (ACS grade, Echo, 99.9%), and tetrahydrofuran (THF) (inhibitor free, high purity, Tedia, 99.8%) were used as received. Nafion D2020 (Coplolymer Resin, 20–22%, Dupont) were also used as received. Electrospun PBz (ES-PBz) fiber mats were prepared based on the procedure described in the work of Parreño *et al.*^21^

### 2.2 Surface modification of electrospun fibers by ozonation

Functional groups of hydroxyls (–OH) were incorporated on the samples' surfaces by free radical ozonation of electrospun fiber mat using Ozonizer (Three Oxygen Ent. Co. Ltd Taipei, Taiwan) at low temperature. Sample of fiber mat (approx. 3 cm × 3 cm) was immersed completely in 400 mL of deionized (DI) water inside the 500 mL glass reactor vessel. Then, the reactor vessel was placed in a basin with water and ice to improve ozone utilization at low temperature. Afterwards, the Ozonizer was switch on and set to the desired operating conditions with the oxygen (O_2_) connecting tube placed inside the reactor vessel to supply the oxygen from the Ozonizer to the reactor vessel containing the sample. The oxygen flow rate was adjusted and maintained at around 8 L min^−1^ to have enough amount of O_2_ bubbling inside the reactor for effective treatment. Ozone treatment of the sample was carried out for 30 min. After completion, the sample inside the reactor was purged with argon for another 30 min to remove residuals. Then, the sample was set aside and dried at room temperature in a Petri dish.

### 2.3 Characterization of surface-modified electrospun fibers

Attenuated total reflectance with Fourier transform infrared spectroscopy (ATR-FTIR) (PerkinElmer Spectrometer, MA, USA) was used to investigate the structural composition of the electrospun PBz fiber sample before and after surface modification by ozone treatment. A portion of the fiber mat sample was directly analyzed in the ATR accessory without any sample preparation. All measurements were performed at the range of 4000–400 cm^−1^ with a resolution of 4 cm^−1^. The changes in surface chemical composition were analyzed by X-ray photoelectron spectroscopy (XPS) (VG Microtech MT-500 ESCA). Resolution of sub peaks was performed using the least-squares peak analysis software, XPS PEAK 95 version 3.0. The surface wettability of surface-modified and pristine ES-PBz fiber mats were measured by water contact angle meter (First Ten Angstroms (FTA) Model: FTA 1000 B) with water drops of about 5 μL at ambient conditions. The contact angle data were obtained from the average of three replicates using five measurements of mat samples.

### 2.4 Preparation of hybrid composite membrane

The fibers as fillers for the hybrid composite were prepared *via* electrospinning and thermally crosslinked as described in the work of Parreño *et a*l.,^21^ prior to composite preparation. Physical blending method was employed in the fabrication of hybrid composite where the polymer was directly poured into the fiber mat to disperse it into the whole surface of the fiber mat, followed by drying to solidify the composite. The amount of Nafion and the time for homogenization and curing were varied for the preparation of composites. The amounts of Nafion dispersion solution used were 6 mL and 9 mL. Before physical blending, the polymer dispersion was homogenized and cured to remove bubbles and prevent interfacial and surface skin defects. The homogenization and curing time used were 90 min for ultrasonication with varied curing time of 1 h and 24 h prior to composite formation. Then, using different amount of Nafion solution that was homogenized and cured, the polymer solution was poured into the fiber mat sample and completely dispersed all throughout the sample. Afterwards, it was set aside for 1 h to let the sample absorb completely the Nafion solution. The hybrid composite sample was dried in the vacuum oven (Deng Yng, DOV 300, Taipei, Taiwan) at 40 °C, and 50 °C, each for 1 h, then 80 °C, 90 °C and 105 °C, each for 0.5 h. Then, the sample was cooled down. After cooling, the composite was immersed in DI water overnight to further remove residual solvents.

### 2.5. Interfacial compatibility of ozone-treated fibers with Nafion

The surface morphology and structure were analyzed using Scanning Electron Microscope (SEM) (Phenom XL, Thermo Fisher Scientific, AZ, USA) to examine the interfacial adhesion and compatibility of fibers with Nafion matrix in the final hybrid composite. The fibers as reinforcing fillers were examined for its ability to blend with Nafion in terms of surface appearance as indicated from the resulting skin and densification of the composite.

## 3. Results and discussion

### 3.1 Structural composition and surface chemistry of ozone-treated PBz fibers

**Fig. 1:**
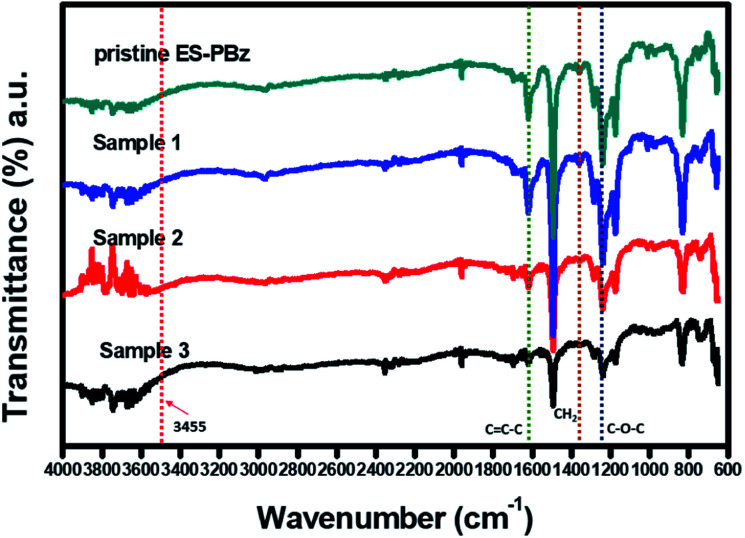
Spectroscopy results of pristine ES-PBz fibers and ozone-treated ES-PBz fibers (samples 1, 2 and 3).

Electrospun PBz fibers were first characterized for its structural composition to determine if there were changes in the chemical structure of the PBz after undergoing the surface modification *via* ozonation. Attenuated total reflectance (ATR) as sampling technique in conjunction with Fourier transform infrared (FTIR) spectroscopy was used to analyze the characteristic absorption peaks of the PBz fiber sample to correlate spectral change with corresponding modifications in surface structure and chemical composition of the samples. As shown in Fig. 1, the spectra of the pristine electrospun PBz fibers showed the characteristic peaks associated with the benzoxazine structure at 1230–1235 cm^−1^ (asymmetric stretching of C–O–C), at 1330–1340 cm^−1^ (CH_2_ wagging into the closed benzoxazine ring) and at 1495–1510 cm^−1^ (tri substituted benzene ring). These absorption peaks were also present in the spectra of samples that were modified by ozonation. Three samples (1, 2 and 3) of ozone-treated ES-PBz fibers as shown in Fig. 1 were analyzed for their characteristic absorption peaks to better evaluate the spectral changes that occurred after ozonation treatment. Based on these results, there were no additional peaks in the spectra of the three samples which indicated that no other interactions occurred when the PBz fibers undergone ozone treatment.

**Fig. 2:**
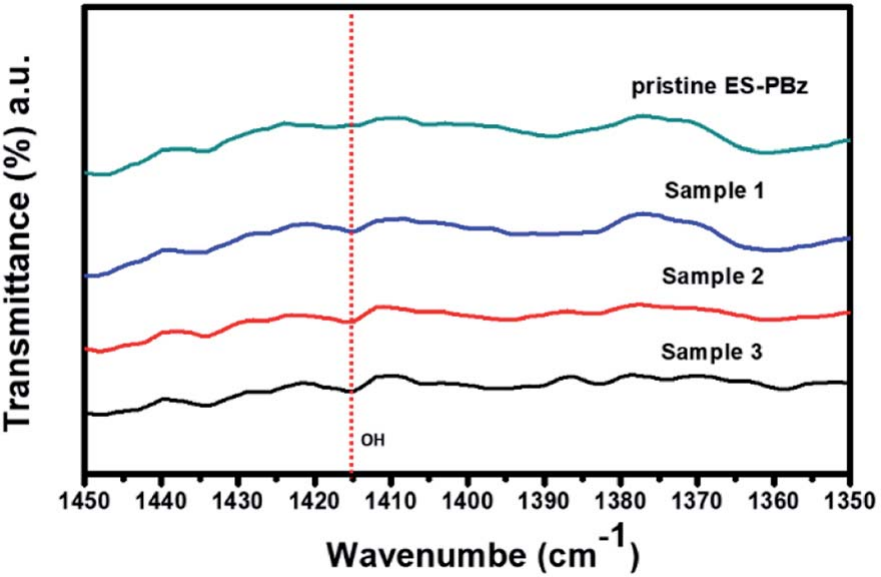
IR spectra of –OH bend at wavenumber of 1415 cm^−1^.

To further examine the results of surface modification and confirm the endowed –OH functional groups, the spectra were analyzed for both the pristine PBz fibers and ozone-treated PBz fibers. The hydroxyl groups incorporated onto fibers' surfaces have the potential to enhance the water retention capability of the fiber mat when used as fillers in the Nafion composite. According to Ketpang *et al.*,^22^ the peaks at wavenumber of 3455 and 1625 cm^−1^ corresponds to the –OH stretching vibration and –HOH bending vibration, respectively. However, based on the result at wavenumber of 3455 cm^−1^, the peak of –OH band was not present in the IR spectra of surface-modified samples which could be due to the limitation in spectral resolution of the IR. Although the broad band for the –OH stretching vibration as additional functional group in the fibers' surfaces was not shown in the spectra, another way to examine the result was the –OH that formed as –COOH due to the electrophilic aromatic substitution of –OH in the C–H as adsorption sites or active sites of benzene ring in PBz. Based on a larger image of the IR spectra in Fig. 2, it revealed the –COOH that formed after ozonation which produced a peak of –OH band at 1415 cm^−1^ as the hydroxyl bonded to the PBz's aromatic ring. This confirmed the presence of hydroxyl groups onto surfaces of the PBz fibers.

**Fig. 3:**
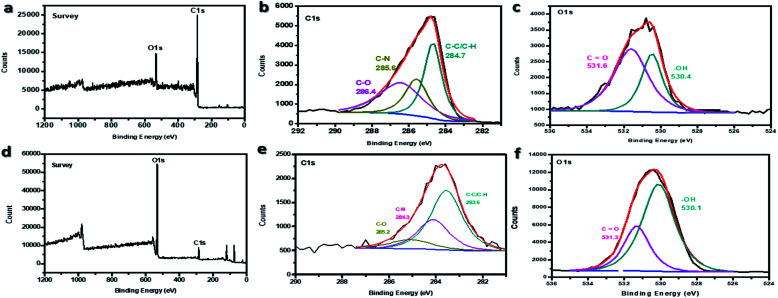
XPS spectra of (a) pristine PBz fibers and (d) ozone- treated PBz fibers with the C 1s and O 1s peak-fitting curves of (b and c) pristine PBz fibers and (e and f) modified PBz fibers.

The further confirmation on surface modification of ES-PBz fibers in relation to –OH functional groups was examined by X-ray photoelectron spectroscopy (XPS). As shown in the XPS survey scans of the samples in Fig. 3, the two main elemental components, carbon (C) and oxygen (O), were present in the wide spectra of the samples for both pristine PBz fibers and ozone-treated sample. The survey scans in Fig. 3a and d showed that the O/C content ratio of ozone-treated PBz fibers was higher than the pristine PBz fibers which indicated that the electrospun fibers were successfully oxidized. The C 1s peaks for both samples were enlarged into narrow peaks in Fig. 3b and e. These peaks were deconvoluted into three separate peaks indicating the carbon-containing group corresponding to the observed bond energies. The O 1s peaks for both samples were also deconvoluted into two separate peaks as shown in Fig. 3c and f to validate the higher O/C content ratio after ozonation of the PBz fibers. The scans of O 1s confirmed the same findings in the O/C content ratio of the C 1s scans.

The percentage of each group was calculated according to the fitting peak area. The calculated compositions were 80.5% O and 19.5% C for ozone-treated sample while only 16.9% O for pristine PBz fibers with higher composition of 83.1% C as shown in Table 1. This result validated the surface modification *via* ozonation of PBz fibers which was in agreement with the results of IR spectroscopy.

**Table 1:** Elemental compositions of pristine PBz fibers and ozone-treated PBz fibers

At%	Pristine PBz fibers	Ozone-treated PBz fibers
C 1s	83.1%	19.5%
O 1s	16.9%	80.5%

### 3.2 Surface wettability of ozone-treated PBz fibers

**Fig. 4:**
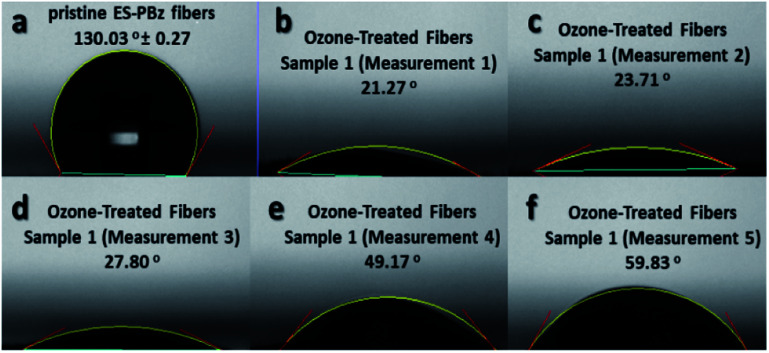
Water contact angle of (a) pristine PBz fibers and (b–f) ozone-treated PBz fibers of one sample.

The measurement of water contact angle is another way to assess the surface chemistry of the samples after surface modification, if there have been changes in the wettability of the samples. As shown in Fig. 4, the water contact angles were reduced for the surface-modified samples which validated the surface chemistry results of XPS that confirmed the surface modification. Electrospun PBz fibers are hydrophobic with water contact angle of 130.03° ± 0.27 (Fig. 4a) but after the modification it became more hydrophilic with water contact angle ranging from 21.27° to 59.83° (Fig. 4b–f) for the five measurements of one sample. These results indicated that the surface was modified by hydrophilization through electrophilic aromatic substitution in the benzene ring with –OH functional groups from ozone treatment.

### 3.3 Preparation of hybrid composite of PBz fibers with Nafion

**Fig. 5:**
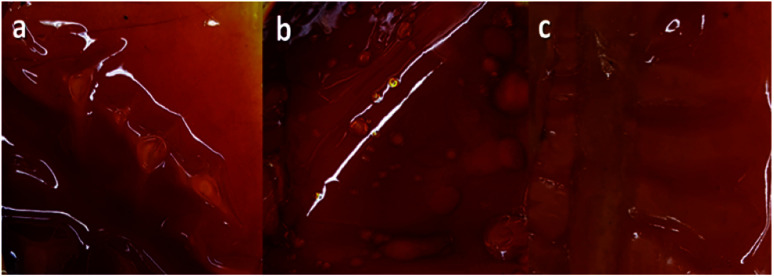
Hybrid Composite with Electrospun PBz Fibers as Fillers prepared with (a) 9 mL of Nafion, homogenized at 90 min and 1 h curing time, (b) 6 mL, homogenized at 90 min and 1 h curing time and (c) 6 mL of Nafion, homogenized at 90 min and 24 h curing time (scale 1 : 2 cm; magnified 2×).

The two factors investigated in the preparation of composite with fibers were the amount of Nafion dispersion solution and homogenization and curing time. These factors significantly influence the compatibility and interfacial adhesion based on the surface morphology and structure of the composite. Initially, the preparation of Nafion solution involved homogenization by ultrasonication for 90 min. Then, set aside for 1 h to further dispersed and removed the bubbles before pouring onto the PBz fiber mat. The amount of Nafion dispersion solution used in the first trial was 9 mL for the fiber mat sample with approximate size of 3 cm × 3 cm. After drying of the final composite in the vacuum oven, it appeared to have uneven, wrinkled surface with bubbles present in the composite as shown in Fig. 5a. Then, based on the skin appearance, the amount of Nafion solution was reduced to 6 mL for the same size of fiber mat sample in the second trial. But homogenization time was retained at 90 min for ultrasonication and followed by 1 h curing time. There was a slight improvement observed in the surface appearance of the composite as compared to previous trial as shown in Fig. 5b. The presence of bubbles was still observed but with smoother and less wrinkled composite's skin. For the third attempt, the same amount of Nafion solution was used with homogenization by ultrasonication of 90 min but set aside for longer curing time of 24 h before it was poured onto the fiber mat sample. It was observed that the composite produced at this condition showed smoother and even surface without the presence of bubbles and lesser prominent wrinkles in the composite's skin as shown in Fig. 5c.

These results showed that reducing the amount of Nafion dispersion solution from 9 mL to 6 mL was enough to completely embed the fibers without affecting the preparation process. It had similar effects on surface morphology and structure with the presence of bubbles and wrinkles. However, allowing longer time for dispersion and curing of Nafion solution after sonication prior to use in the composite preparation, resulted to better surface appearance as exhibited in Fig. 5c.

**Fig. 6:**
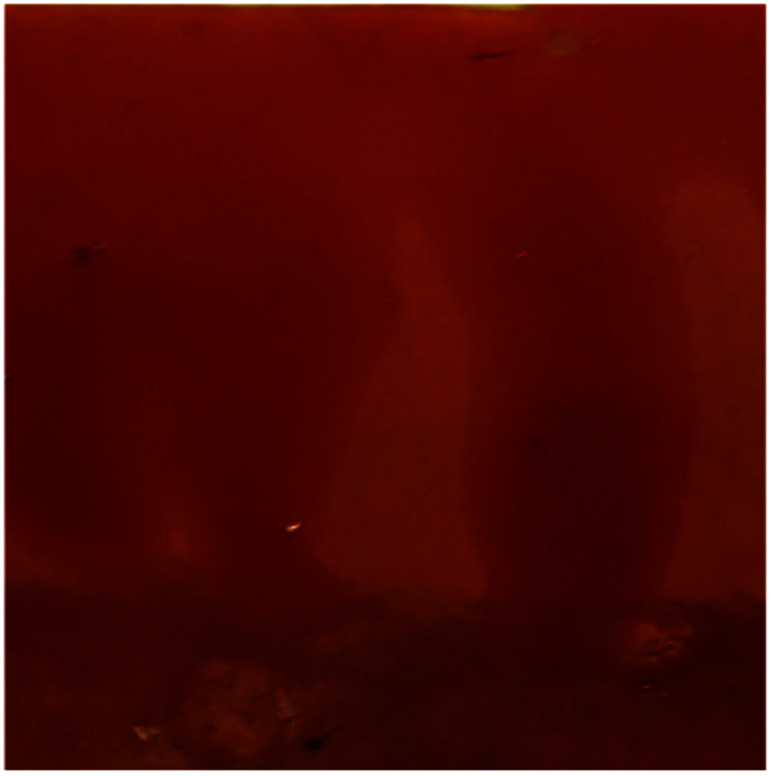
Final Hybrid Composite of Nafion with Electrospun PBz Fibers (scale 1 : 3 cm; magnified 3×).

Thus, in the final preparation of composite of Nafion with added fibers as shown in Fig. 6, the adjusted processing parameters of 6 mL Nafion solution and 90 min sonication followed by 24 h curing time were applied in producing the final hybrid composite.

### 3.4 Compatibility and interfacial adhesion of PBz fibers with Nafion

**Fig. 7:**
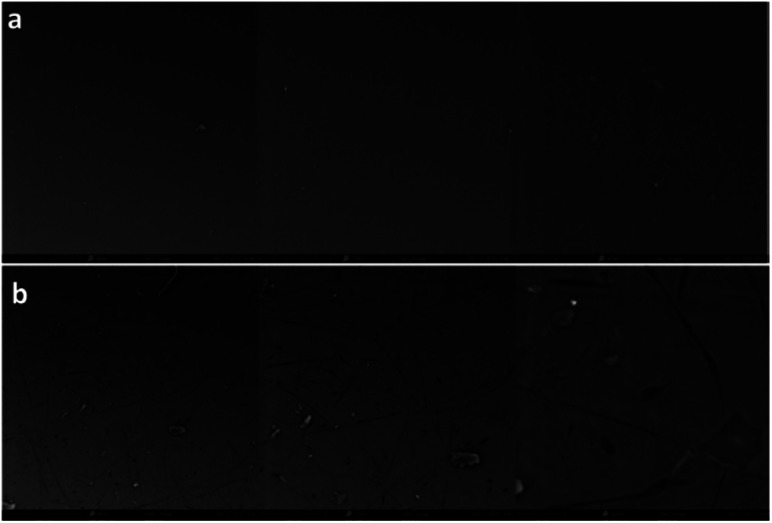
SEM images of (a) pure Nafion and (b) hybrid composite of Nafion with ozone-treated fibers; (magnification: 800, 1350 and 5000×; scale bar: 100, 50 and 10 μm).

Analyzis of the SEM images of the composites were conducted to determine the compatibility and interfacial adhesion between the ozone-treated fibers as fillers and Nafion as polymer matrix in forming the hybrid composite. The surface morphology as well as the skin of the composite were considered as important indicators of well-prepared hybrid composite based on SEM images. The irregularities such as voids, flaws, fractures, lines, and defects were also checked to show differences in the skin appearance. For the ozone-treated fiber-reinforced composite as shown in Fig. 7b, the fibers were indistinguishable from the structure which showed that Nafion was able to completely penetrate between the fibers and formed a continuous phase within the composite. This structure was comparable with the skin of pure Nafion without ozone-treated fibers in Fig. 7a. Based on the work of Sigwadi *et al.*,^23^ the result was the same with the SEM surface morphology of Nafion 117 having a skin of dark in color and plain surface due to absence of additives or fillers. According to Prykhodko *et al.*,^24^ interfacial defects arise from the existence of the space charge layer in addition to the material that fills the polymer space. But it showed that the composite was fully densified with Nafion which was an indication of good interfacial adhesion and compatibility between the resin matrix and fibers without any effect from the space-charged layer or from the hydroxyl in the fibers' surfaces. This also confirmed that Nafion adhered firmly to the fiber surface and no evident detachment was observed at the Nafion/fibers interfaces. Although the composite showed densification, slight defects were observed in the skin with the presence of numerous tiny shallow lines. Based on the magnified images, the shallow lines were not fractures or cracks in the composite's structure but were just visible on the composite's surface.

## 4. Conclusions

Nafion is the widely-used commercial membrane and remains to be the standard polymer electrolyte membrane for PEMFC. Many research efforts have been conducted in the last five years to further enhance its properties and address the current limitations of Nafion for fuel cell application. New routes for enhancement were done such as chemical modification of functional properties as well as physical modification by additive enhancement. One of the most promising modification methods is by incorporating fibrous structures as fillers to produce hybrid composite with Nafion. This study utilized the second route by incorporating electrospun fibers as fillers with Nafion by investigating the compatibility and interfacial adhesion. The main purpose was to provide enhancement in water transport and hydration of the composite through the effect of surface-modified properties of electrospun fibers. The ozonation treatment of electrospun PBz fibers resulted to potentiated oxygen-based functional groups of hydroxyl radical onto fibers' exposed surfaces. The presence of higher oxygen-based functional groups in fibers' surfaces was correlated to the change in surface wettability by becoming hydrophilic from a hydrophobic pristine PBz fibers. In the preparation of hybrid composite, the processing parameters that showed significant effects were lesser amount of Nafion but longer curing time prior to composite formation. The production of final hybrid composite proved the compatibility of fillers and polymer matrix. The composite was fully densified with Nafion which indicated good interfacial adhesion and compatibility between the resin matrix and fibers without any effect from added hydroxyl in the fiber's surfaces. This also confirmed that the Nafion adhered firmly to the fiber surface and no evident detachment was observed at the Nafion/fibers interfaces. Thus, ozonation can be carried out on crosslinked PBz fibers by enhancing its target properties and then utilized as fillers for Nafion in a composite. This outcome could be used as basis to further study the succeeding route as a result of ozonation by looking into the next step for potential surface-initiated grafting onto the fibers. This could provide additional functional groups aside from hydroxyls such as sulfonates for polymer electrolyte membrane.

## Author contributions

Conceptualization, R. P. P. J. and A. B. B.; methodology, R. P. P. J.; formal analysis, R. P. P. J. and A. B. B.; investigation, R. P. P. J.; data curation, R. P. P. J.; resources, R. P. P. J. and A. B. B.; writing – original draft preparation, R. P. P. J.; writing – review and editing, R. P. P. J. and A. B. B.; funding acquisition, R. P. P. J. and A. B. B.

## Conflicts of interest

There are no conflicts to declare.

## Supplementary Material
